# A Manual of the Diseases of the Camel, Etc.

**Published:** 1891-08

**Authors:** 


### REVIEWS.

A MANUAL OF THE DISEASES OF THE CAMEL, AND OF HIS MANAGEMENT AND USES. By John Henry Steel, V.S., A.V.D., F.R.C.V.S., F.C.S., Fellow of the University of Bombay, Principal and Professor of Veterinary Science, Bombay Veterinary College, Etc. Illustrated. Madras: G. W. Taylor. 1890.

This interesting work on the camel by Dr. Steel, of Bombay, possesses not only the value that attaches to a well written scientific monograph on a subject of the highest importance, but is especially serviceable to those who superintend the practical working of zoological collections, for the reason that it dispels many prevalent and pernicious impressions concerning the uses and management of the strange animal. To the denizen of the desert he is an undeniable blessing—he is no less dear than the barb of Araby, the idol of the Eastern household—and he is cared for with the utmost solicitude; nor need we not wonder at this, because in the hands of his wild and untutored master he is managed with that success which unlimited experience has furnished, and hence is the very ideal of a beast of transport. For them he is the true ship of the desert; and to his docility, sagacity, and inexhaustible patience whole families owe their lives amid the burning sands of the desert. A simple lariat guides his steps, he quickly heeds the voice of his master, and gives timely admonition of the impending storm; but, in the hands of the ordinary British soldier who can be taught nothing beyond what the guard-house has drilled into him, the camel is altogether a different animal. In the Soudan and Afghanistan he has proved a dismal failure as a transport because he was handled by those who knew little or nothing about his habits, his necessities, his power of endurance, and his weak points. Under the impression that he contains within him a perennial well-spring of pure water he has been allowed to go for weeks without water, on forced marches, till he dropped by the wayside, dying of thirst. The camel is not a tank; moreover, his ignorant and cruel army-masters scarcely ever think of grooming him or removing from his sore and festered back at night, after a wearisome march under a broiling sun, the galling pack-saddles. It is no wonder that such men complain of the camel as a vicious and irritable brute, mangy and malodorous.

The services of the camel have been, to a great extent, superseded in Asiatic Russia by railroads; but on the other hand, he has been lately introduced into countries that had not known him before, and here he is highly prized. Satisfactory experiments, made by the U. S. Government, have proved that the two-humped camel can be made to render excellent services in the warm climate of the Southern states. Quite a number of them are at present employed in the South for ordinary transportation purposes, and there can be no doubt that he will prove wonderfully valuable in helping to open up the interior of Australia. An atmosphere laden with humidity is not a favorable condition for camel life and, in selecting localities for his services, this fact should be borne in mind. Hence the dry interior of sandy regions like the Central Asian desert are best suited for the exhibition of his powers, as a beast of burden. His whole anatomical structure clearly proves his fitness for a life on the sandy plains; he possesses wonderful endurance, and this quality is inestimable when we consider the conditions under which the services of the camel are called in action. Dr. Steel insists that in respect to this quality, Col. Burnaby was greatly mistaken when he spoke of the camel as "very delicate beast of burden". The fact that he is prone to disease is but an expression of the harsh treatment to which he is frequently subjected, and the little cure that is bestowed upon him when suffering. In point of endurance he is even superior to the mule, with which animal he is an unwilling competitor as a transport. He possesses the same amplitude of chest and the same narrowness of belly—conditions obviously favorable to wind and untiring effort; and then he even surpasses the mule's celebrated congener that feeds on thistles, by the ease with which he stows away hard prickly plants like cactus leaves: but it is his long limbs and spreading foot pads that especially render the camel an ideal beast of burden and of transport. It is for this reason that Gen. Wolseley recommends that camels be first sent over a designated line of march that they may consolidate the soft and slippery soil by a packing process.

Notwithstanding the popular belief that the camel can endure thirst for an indefinite period, still it is important that he be watered once every twenty-four or forty-eight hours, as the privation of water for a longer time cannot but prove injurious. This is so well understood by the Arab-master of the camel that he will water his beast daily except when about to undertake an unusually long journey, and then he prepares him for a protracted thirst by forcing five or six additional gallons of water down his throat. It is previous to such long journeys through the desert that every precaution should be taken in order to ensure satisfactory service, and the failure of the camel to furnish such results when employed as an army transport by the British government in the East, has been entirely due to the neglect of such precautionary measures as Arabian experience has proved to be indispensable.

Dr. Steel dwells on the principal feature of preparation for long journeys at length, and gives such directions in a straightforward and practical manner that there can be no excuse henceforth for throwing the blame of failure on the poor patient Bactrian. According to Dr. Steel efficient grooming is one of the most important preliminary steys to a long journey and the one which is most frequently neglected. This accomplished, a thorough inspection of every part of the body should be made and a satisfactory condition of the feet should be especially ascertained. Important directions, too, are given concerning the manner of loading the camel, the character and make-up of the *Pulàn* or pack-saddle, and its proper method of adjustment. The full understanding of these details is especially important at the present time, when the field of service of the camel is being greatly enlarged and embraces a portion of the United States.

Here and in Australia the study of Dr. Steel's monograph cannot but turn out to be of the highest practical utility. The chapter entitled "General Consideration on Diseases of the Camel" furnishes the key to the special pathology of the bacterian family and is replete with valuable information. From a perusal of this chapter it will be gathered that the majority of cameline diseases are the result of mal-treatment and neglect. The exceeding prevalence of abscesses and the marked tendency of wounds to suppurate is plainly the result of such a cause. Though the natives know better how to handle a camel when in good health than the average European; it is lamentable true that the poor beast could fall into no worse hands when disease becomes his lot. Then the most absurd measures are employed by these rude pathologists of the wilds, and death speedily follows sickness. Consequently, cameline therapeutics and materia medica offer a wide field for much needed observation and experience, and what Dr. Steel submits to his reader's attention in this respect is of great practical interest. One of the most fatal disorders from which the camel suffers is Anthrax, and hundreds perish from this disease annually. Unfortunately it is a most intractable malady, though Dr. Steel offers some rational suggestions as to the best method of treating it. He recommends especially that Pasteur's system of Anthrax-inoculation be enforced by State governments, and so an effort be made to stamp it out.

Though cameline pathology and therapeutics may be said to be still in their infancy, Dr. Steel has proved a valuable pioneer in a new and more enlightened order of things and a careful study of his work should be made by all those interested in the subject.

C. M. O'L.

